# Influence of CD38/PD1 co-expression on T cell subsets in HIV-TB co-infection

**DOI:** 10.1186/1471-2334-12-S1-O4

**Published:** 2012-05-04

**Authors:** Sharada Ramaseri Sunder, Surekha Rani Hanumanth, Anuradha Bandaru, Satya Sudheer Pydi, Sumanlatha Gaddam, Subbanna Jonnalagada, Vijaya Lakshmi Valluri

**Affiliations:** 1 LEPRA India – Blue Peter Public Health & Research Centre, Cherlapally, Hyderabad, India; 2 Department of Genetics, Osmania University, Hyderabad, India; 3 Bhagawan Mahavir Medical Research Centre, Hyderabad, India

## Background

Excessive immune activation as indicated by CD38 expression is a characteristic feature of HIV progression. There are growing evidences suggesting that immune inhibitory signals such as PD1 expression also play an important role in progression of the disease. However, the relationship between these positive (CD38) and negative (PD1) immune signals on T cells in HIV-TB co-infection has not been studied in detail so far.

## Methods

Expression levels of CD4, CD8, CD38 and PD1 were analyzed in peripheral blood collected from HIV, TB, HIV-TB and healthy controls using standardized protocol on flow cytometer.

## Results

The percentage CD8+/CD38+ and CD8+/PD1+ cells was high in HIV-TB and HIV when compared with control and TB, while there was no significant difference between HIV and HIV-TB. The CD38+/PD1+ co-expression was significantly high (p<0.01) on CD8+ cells in HIV-TB when compared with HIV, control and TB groups, inferring that CD38/PD1 phenotype distinguishes CD8 T-cell responses between HIV and HIV-TB co-infection.

## Conclusion

High CD38+/PD1+ co-expression on CD8 cells probably causing a variation in CD8 cell responses could perhaps be a risk factor for development of tuberculosis in HIV-positive individuals. The use of CD8/CD38/PD1 markers for HIV-TB needs contemplation.

